# Altered Expression of Transmembrane Mucins, MUC1 and MUC4, in Bladder Cancer: Pathological Implications in Diagnosis

**DOI:** 10.1371/journal.pone.0092742

**Published:** 2014-03-26

**Authors:** Sukhwinder Kaur, Navneet Momi, Subhankar Chakraborty, David G. Wagner, Adam J. Horn, Subodh M. Lele, Dan Theodorescu, Surinder K. Batra

**Affiliations:** 1 Department of Biochemistry and Molecular Biology, University of Nebraska Medical Center, Omaha, Nebraska, United States of America; 2 Department of Pathology and Microbiology, University of Nebraska Medical Center, Omaha, Nebraska, United States of America; 3 Buffett Cancer Center, Eppley Cancer Institute, University of Nebraska Medical Center, Omaha, Nebraska, United States of America; 4 University of Colorado Comprehensive Cancer Center, Aurora, Colorado, United States of America; UCSF/VA Medical Center, United States of America

## Abstract

**Purpose:**

Radical changes in both expression and glycosylation pattern of transmembrane mucins have been observed in various malignancies. We and others have shown that MUC1 and MUC4, two transmembrane mucins, play a sentinel role in cell signaling events that drive several epithelial malignancies. In the present study, we investigated the expression profile of MUC1 and MUC4 in the non-neoplastic bladder urothelium, in various malignant neoplasms of bladder and in bladder carcinoma cell lines.

**Material and Methods:**

Immunohistochemistry was performed on tissue sections from the urinary bladder biopsies, resection samples and tissue microarrays (TMAs) with monoclonal antibodies specific for MUC1 and MUC4. We also investigated their expression in bladder carcinoma cell lines by RT-PCR and immunoblotting.

**Results:**

MUC1 is expressed on the apical surface or in umbrella cells of the normal non-neoplastic bladder urothelium. Strong expression of MUC1 was also observed in urothelial carcinoma (UC). MUC1 staining increased from normal urothelium (n = 27, 0.35±0.12) to urothelial carcinoma (UC, n = 323, H-score, 2.4±0.22, p≤0.0001). In contrast to MUC1, MUC4 was expressed in all the layers of non-neoplastic bladder urothelium (n = 14, 2.5±0.28), both in the cell membrane and cytoplasm. In comparison to non-neoplastic urothelium, the loss of MUC4 expression was observed during urothelial carcinoma (n = 211, 0.56±0.06). However, re-expression of MUC4 was observed in a subset of metastatic cases of urothelial carcinoma (mean H-score 0.734±0.9).

**Conclusion:**

The expression of MUC1 is increased while that of MUC4 decreased in UC compared to the normal non-neoplastic urothelium. Expression of both MUC1 and MUC4, however, are significantly higher in urothelial carcinoma metastatic cases compared to localized UC. These results suggest differential expression of MUC1 and MUC4 during development and progression of bladder carcinoma.

## Introduction

Bladder cancer (BCa) is the fifth common malignancy in the United States accounting for nearly 72,570 new cases and 15,210 cancer-related deaths during 2013 [1]. The urothelial carcinoma (UC) is the most common histologic type of BCa that accounts for >90% of the newly diagnosed cases. UCs at the time of diagnosis range from superficial low-grade papillary lesions (associated with better prognosis) to highly invasive malignant carcinomas (highly aggressive with a low survival). Approximately, 70–80% of newly diagnosed UCs are “non–muscle invasive” wherein the disease is confined to the bladder mucosa or lamina propria (stage Ta/T1 according to TNM classification) [2]. About 10%–30% of these tumors advance to “muscle-invasive disease” (“high grade” UC) (stage T2/T3) [2]. Low-grade papillary cancers are generally non-invasive (only <15% invade the bladder wall) and thus amenable to surgical resection. However, the cases of high grade invasive carcinoma are associated with high probability of metastasis and mortality [3], [4]. Cytology and cystoscopy with tissue biopsy remain the most accurate methods available to detect BCa till date. Cytology is highly specific for high-grade urothelial carcinoma but not for low-grade urothelial carcinoma.

In recent years, aberrant changes in the expression and glycosylation of mucins have been reported in inflammatory, premalignant and malignant conditions [5]–[10]. Mucins are glycoproteins that are characterized by the presence of high degree of O- and N-glycosylation together with highly repetitive short stretches of amino acid residues (termed as “tandem repeats”) [9]. These are broadly divided into two categories namely membrane bound mucins and secreted/gel forming mucins. Importantly, MUC1 and MUC4 represent the well characterized trans-membrane mucins playing important roles in cellular physiology. By virtue of their structure and biochemical composition these mucins participates in lubrication and hydration of cell surfaces, protection from microorganisms (bacteria and viruses) and degradative enzymes [11]. Variation in the expression and glycosylation pattern of MUC1 and MUC4 has been observed in several epithelial malignancies including pancreatic, breast, colon, prostate and lung cancer [7], [9], [12], [13]. They have been shown to play a critical role in tumor growth, intracellular and extracellular signaling, tumor–stromal interactions, metastasis, and resistance to chemotherapeutic agents and in immune surveillance [7], [11], [12], [14]. The availability of highly specific reagents (*e.g.* monoclonal antibodies), some capable of recognizing altered glycoforms has made mucins attractive targets for the early diagnosis of epithelial malignancies. Altered expression and localization pattern of MUC1 have been observed during progression of malignant neoplasms of bladder [15]–[18], however to date there is a dearth of information on the status of MUC4. Considering their protective and lubricating roles, it is important to assess their functions in the healthy bladder and the alteration in their expression during the development and progression of urothelial carcinoma.

In the present study, we examined the expression patterns of MUC1 and MUC4 in non-neoplastic bladder urothelium and malignant neoplasms of bladder tissues on tissue microarrays (TMA) and tissue sections from the urinary bladder biopsies and resected samples. Further, the correlation between expression of these mucins and tumor grade (grades 1–4) was examined to define the diagnostic and physiological significance of TM mucins during pathogenesis of UC.

## Materials and Methods

### Tumor Cell Lines and Tissue Specimens

Archived formalin fixed BCa tissue samples were obtained University of Nebraska Medical Center tissue bank. The review board found this study, with its de-identified database, to be exempt from institutional review board review. Bladder TMA were purchased from US Biomax for MUC1 staining (cat#BL802), Accumax for MUC4 (cat#A215II) and Bladder TMA (2004) (a kind gift from Dr. Henry Frierson from University of Virginia Charlottesville VA). IHC staining was performed using mAb HMFG2 (anti-MUC1 antibody, a kind gift from Dr. Gendler, Mayo Clinics), and mAb8G7 (anti-MUC4 antibody, generated against tandem repeat peptide, STGDTTPLPVTDTSSV in our laboratory and reactive with both native protein and peptide) [19]. Bladder carcinoma cell lines [T24 and HT1376, (derived from bladder transitional cell carcinoma), TCCSUP (derived from an anaplatic transitional cell carcinoma) SCaBER (derived from bladder squamous cell carcinoma) were maintained in the American Type culture Collection (ATCC) specified culture media supplemented with 10% FBS and 1% penicillin-streptomycin (Gibco BRL, Grand Island, NY). Growth media was changed on alternate days and the cells were trypsinized at 70% confluency.

### Immunohistochemistry (IHC)

For analyzing MUC1 and MUC4 expression in malignant neoplasms of bladder, IHC was performed as described previously on TMA and tissue sections of samples from the urinary bladder [7]. Biopsy and resection samples of urothelial carcinoma were reviewed by the pathologists (Drs. S Lele, AH Horn, and DG Wagner at UNMC) to confirm the diagnoses and also select representative blocks for immunohistochemical analyses. For tissue IHC, 4 μm thick sections were cut from paraffin-embedded tissue blocks using tissue microtome and were adhered on to the charged glass slides. Bladder TMA and tissue sections were incubated overnight at 56°C followed by deparaffinization using repeated washes of xylene (5 min each) followed by rehydration of tissues with graded alcohol. After rehydration, tissues were incubated with methanolic solution of 3% H_2_O_2_ for quenching of endogenous peroxidase activity. Heat induced antigen retrieval was performed heating the sample in 0.01M citrate (pH 6.0, 95°C) buffer for 15 min in microwave oven. Following antigen retrieval, the sections were blocked with horse serum (ImmPRESS Universal antibody kit; Vector Laboratories) for 3 hrs at room temperature. The slides were further incubated with anti-MUC1 (HMFG2 at 1∶5 dilution of enriched supernatant in PBS), MUC4 antibody 8G7 (1∶2000 dilution of a 1.8mg/ml stock in PBS) [20] overnight at 4°C. After four washes with PBS-T (PBS containing 0.05% Tween 20), the sections were incubated with anti-mouse secondary antibody (ImmPRESS Universal antibody kit; Vector Laboratories) for 30 min. and subsequently, the color was developed by adding 3,3′-diaminobenzidine solution (DAB substrate kit; Vector Laboratories). Reddish brown precipitate indicated positive immuno-reactivity. The slides were counter-stained with Gill’s haematoxylin (Vector Laboratories), dehydrated in graded ethanol followed by xylene and mounted with Vecta-mount mounting medium (Vector Laboratories). All slides were analyzed using a Nikon Eclipse E400 Microscope (Nikon Corporation, Tokyo, Japan).

Each tissue core was evaluated by the uropathologists at UNMC to assess the presence of tumor and scoring of staining. Briefly, each spot on the tissue array was graded based on the intensity of staining on a scale of 0 to 3 (0-no staining, 1-weak staining, 2-intermediate staining, and 3-strong staining). The percentage of positive cells with a given intensity for each sample was determined by the pathologists. The H-score [range: 0 (no staining in any cell) to 3 (all cells staining with the highest (3) intensity] was calculated as the summation of the product of staining intensity (range: 0–3) and proportion of cells staining (range: 0–1; 0-no cell stained and 1–100% cell stained). As per H-score focal reactivity corresponds to H score of ≤0.1, mild reactivity (H score >0.1 but less than or equal to 1.0), moderate reactivity (H score >1.0 but ≤2.0) and intense reactivity (H score >2.0). In case of tissue biopsies due to limited number each tissue type (benign, carcinoma in-situ and carcinoma) present on the slide was scored on the intensity score only. In metastatic cases, sections were scored for H-scoring similar to tissue spots.

### Statistical Analyses

Data was analyzed using Medcalc for Windows version 9.6.4.0 software. For the purpose of analysis, each spot was considered as an individual sample. Intensity of mucin expression were considered as continuous variables, however the type of pathology [UC, squamous cell carcinoma (SCC), adenocarcinoma or papillary carcinoma], grade of cancer were considered as categorical variables. A p value of <0.05 was considered significant.

### Isolation of Total RNA and Reverse Transcriptase-Polymerase Chain Reaction

Cellular RNA from the cultured cells was extracted by RNeasy kit according to manufacturer instructions (Qiagen, Hilden, Germany). Total RNA was quantified using an ultraviolet spectrophotometer (Peqlab ND-1000; Peqlab, Erlangen, Germany) and the quality and integrity of samples were assessed on a 1.5% agarose gel. 2 μg of total RNA from each BCa cell line was reverse transcribed using the first-strand cDNA synthesis kit (Perkin Elmer, Branchburg, NJ) according to the manufacturer’s instructions. The quantitative real time PCR was performed for MUC1, MUC4, and β-actin using following primer pairs: MUC1 (forward primer 5′-GAACTACGGGCAGCTGGACATC′-3′ and reverse primer 5′-GCTCTCTGGGCCAGTCCTCCTG-3′), MUC4 (forward primer 5′-CGCGGTGG TGGAGGCGTTCTT-3′ and reverse primer 5′-GAAGAATCCTGACAGCCTTCA-3′), and β-actin forward primer (5′-TGGACATCCGCAAAGACCTG-3′) and reverse primer (5′-CCGATCCACA CGGAGTACTT-3′) (IDT Cor- alville, IA, USA).

### Western-Blot Analysis

Total protein was extracted from BCa cell lines, resolved and analyzed by immunoblotting according to standard protocol. In brief, cells were washed twice with cold-PBS, scraped in RIPA buffer (100 mM Tris, 5 mM EDTA, 5% NP40, pH-8.0) containing protease inhibitors cocktail (Roche diagnostics, Mannheim, Germany) and allowed to lyse for at least 30 min on ice with intermittent vortexing. Cells were subjected to further lysis by one freeze-thaw cycle and centrifuged at 14,000g for 30 min at 4°C. Supernatants were carefully removed and protein concentrations were determined by Bio-Rad-DC protein estimation kit. For mucin immunoblotting, electrophoresis was performed on 2% SDS-agarose gel using equal amounts of protein samples under reducing conditions. For β-actin, SDS–PAGE (10%) was run under similar conditions. Proteins were transferred to the PVDF membranes and probed with anti-MUC1 (1∶5 diluted mAbHMFG2 supernatant in PBS) and MUC4 (1∶1,000 dilution of mAb8G7) antibodies. After incubation with mouse horseradish peroxidase-conjugated secondary antibodies (Amersham Biosciences Buckinghamshire, UK), signal was detected with an electrochemiluminescence reagent kit (Amersham Pharmacia, Piscataway, NJ).

## Results

In this study, we explored the expression profile of transmembrane mucins (MUC1 and MUC4) in urothelial carcinoma tissue sections and three tissue TMAs. Further, expression levels of these mucins were assessed in bladder carcinoma cell lines.

### MUC1 Expression Analyses during Malignant Neoplasms of Bladder

#### MUC1 expression in the non-neoplastic bladder urothelium

**Figure 1:**
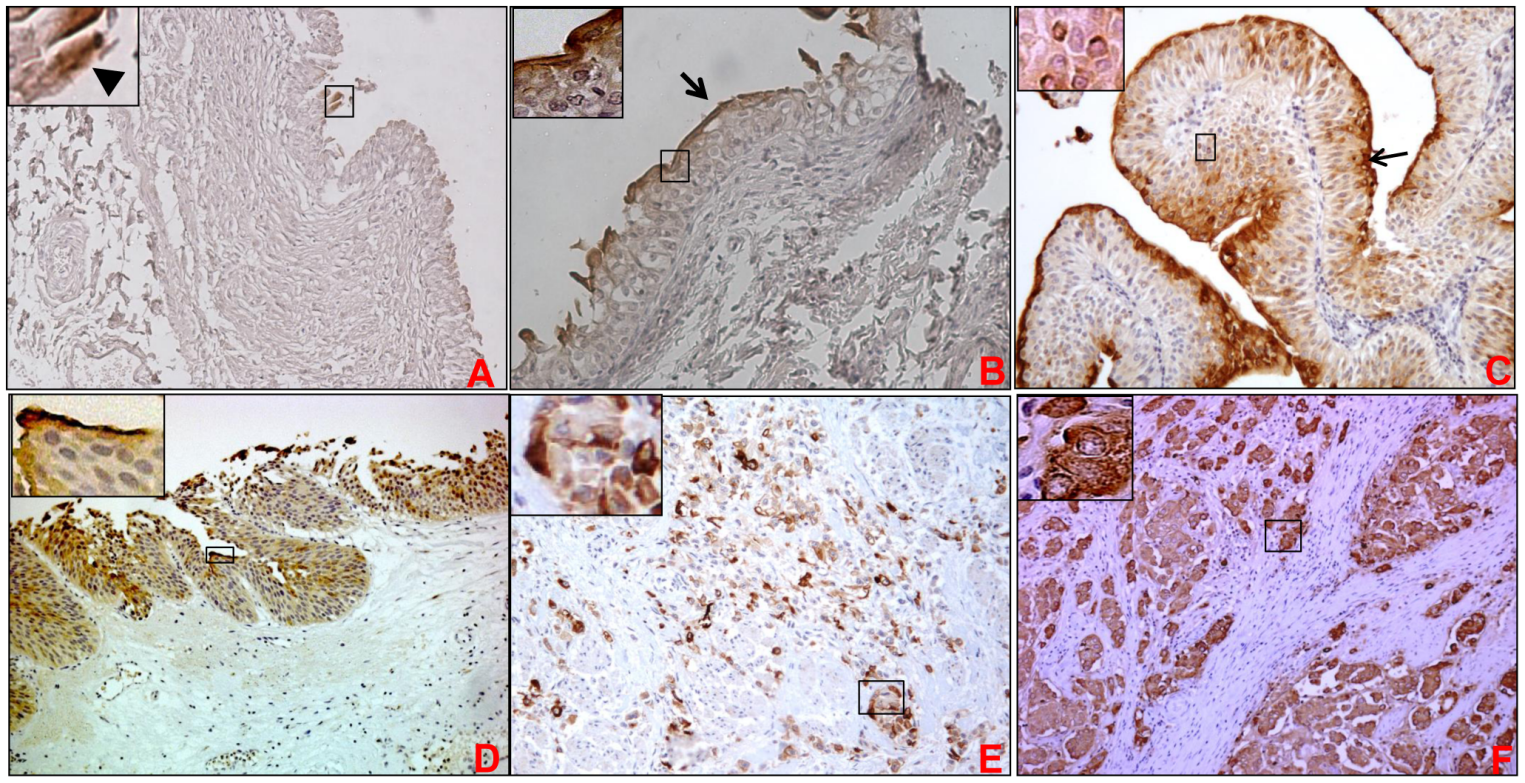
Expression of MUC1 in non-neoplastic bladder urothelium, non-invasive papillary urothelial carcinoma, urothelial carcinoma in situ and invasive high grade urothelial carcinoma. Bladder tumor tissue sections or spots were probed with anti-MUC1 mAb (HMFG2) after non-specific blocking with horse serum. All sections were examined under microscope and the immunoreactivity was evaluated by reddish brown staining. Representative photomicrographs are shown for MUC1 stained non-neoplastic bladder urothelium (A&B), low-grade papillary carcinoma (C), bladder carcinoma in situ (D), and high grade invasive cancer (E&F). In normal bladder urothelium, MUC1 expression was restricted to umbrella cells (shown by arrowhead in panel A and the magnified inset) in majority of samples. In some samples, a sheath of MUC1 mucin was observed over urothelium (arrow in 1B). In low-grade papillary carcinoma, intense staining of MUC1 was observed on the luminal surface of the urothelium and lower intensity of staining was observed in the other layers of urothelium (C). In urothelial carcinoma in situ, MUC1 expression was observed in all the layers of urothelium, however, staining was comparatively stronger in cells closer to the luminal border (D). In invasive high grade urothelial carcinoma, MUC1 staining was observed in cells invading singly (E) or as small groups within the lamina propria (F).

Majority of the non-neoplastic bladder tissues spots from TMA (16/27 or 59%) were negative for MUC1 expression (H score = 0). Minor focal staining was observed in some cases 4/27 (15%). In the remaining spots, mild positivity was observed (7/27 mean H-score 0.58±0.29 on a scale of 0–3) (**Fig. 1A**). Overall, mean H-score of all the cases together was found to be 0.15±0.1 (**Table 1**). In majority of the cases, intense MUC1 staining was restricted to umbrella cells with weak staining in remainder cells of the urothelium (**Fig. 1A** **, arrowhead**). However in few cases, an intense MUC1 staining (4/27 or 15%) was observed superficial layer of the urothelium (**Fig. 1B** **, depicted by arrow**). In general, staining was highly intense in the superficial umbrella cells than in the other layers/cells of the urothelium. A similar finding was noted for tissue sections collected at UNMC which revealed moderate to intense staining for MUC1 (N = 3, mean intensity 1.7±0.58, mean H-score 1.7±0.58) that was restricted to superficial layer or umbrella cells.

**Table 1:** MUC1 expression in non-neoplastic urothelium and various malignant phenotypes of bladder.

Expression of MUC1 in Tissue microarray (TMA)											
	Non-Neoplastic		Urothelial Carcinoma	Metastatic UC		Squamous Cell Carcinoma		Adenocarcinoma		Mucinous Adenocarcinoma	Sarcoma
**N**	27		323	12		51		12		8	7
**Intensity score (Average ± SE)**	0.35±0.12		2.4±0.22^a^^,^ b	1.3±0.23 a ^,^ d		0.52±0.12^a^^,^ b		0.52±0.12 a ^,^ c		0.61±0.27	0.85±0.1
**Intensity (Median)**	0		1.5	0		2.5		1.0		0.5	0
**Intensity score (Range)**	0–2		0–3	0–3		0–3		0–2		0–2	0–3
**H score (mean± SE)**	0.15±0.1		1.59±0.06^a^^,^ b	0.88±0.25^a^^,^ d		2.2±0.15^a^^,^ b		0.43±0.13^a^^,^ d		0.62±0.14 a ^,^ d	0.28±0.05 a ^,^ d
**H-score (Median)**	0		1.5	0.7		2.5		0.4		0.4	0
**H-score (Range)**	0–2		0–3	0–3		0–3		0–2		0–1.5	0–3
**Expression of MUC1 in bladder tissue section**											
		**Non-neoplastic**			**Papillary carcinoma**		**Carcinoma in situ**		**Urothelial Carcinoma**		**Metastatic UC**
**N**		3			7		10		15		28
**Intensity score (Average ± SE)**		1.7±0.58			2.9±0.1		2.86±0.1		3		2.72±0.45
**Intensity (Median)**		1			3		3		3		3
**Intensity score (range)**		1–3			2.5–3		1–3		3		0–3
**H- score (Average ± SE)**		1.41±0.82			2.1±0.83		2.48±0.74		2.43±0.81		1.91±0.15
**H- score (Median)**		1			2		3		3		3
**H-score (Range)**		1–3			0.75–3		0.2–3		0.6–3		0.3–3

a comparison vs. non-neoplastic urothelium.

b p≤0.0001 vs. non-neoplastic urothelium.

c p≤0.005 vs. non-neoplastic urothelium.

d p<0.05.

Urothelial carcinoma (UC); Standard error (SE).

#### MUC1 expression in non-invasive papillary urothelial carcinoma and urothelial Carcinoma In Situ (CIS).

Papillary cancers are generally non-invasive (only <15% invade the bladder wall) and thus amenable to surgical resection. For MUC1, papillary carcinoma cases were restricted to tissue sections alone, as no tissue spot in TMA corresponded to invasive or non-invasive papillary carcinoma. Moderate to intense MUC1 staining was observed in both low and high grade papillary urothelial carcinoma from tissue sections (mean intensity 2.9±0.1 and mean H-score 2.1±0.83 on a scale of 0–3, **Fig. 1C**). Intense staining of MUC1 was observed on the luminal surface of the uroepithelium in low grade papillary urothelial carcinoma. Staining intensity and percentage positivity increased from cells in basal layers to apical layers of the uroepithelium (**Fig. 1C** **, depicted by arrow**). Furthermore, surface layer cells showed more intense staining as compared to the cells underneath uroepithelial layer, demonstrating a non-uniform cell to cell staining pattern (**Fig. 1C**). In case of carcinoma in situ, intense MUC1 staining (mean intensity 2.9±0.1 and mean H-score 2.48±0.74) was observed (N = 10) in the tissue sections (**Fig. 1D**).

#### MUC1 expression in primary and metastatic urothelial carcinoma

In urothelial carcinoma cases, MUC1 staining varied from no reactivity (N = 38 or 12%), focal reactivity (N = 12 or 4%, mean H-score 0.057±0.01) to moderate (N = 147 or 46%, mean H-score 1.02±0.05) and intense immunoreactivity (N = 117 or 37%, mean H-score of 2.86±0.02) (**Fig. 1E and 1** **F**). In invasive cases, MUC1 staining was observed in foci (single or nest of cells) present within the papillary core or in the lamina propria (**Fig. 1E and 1F**), the distribution of MUC1 being both membranous and cytoplasmic.

**Figure 2:**
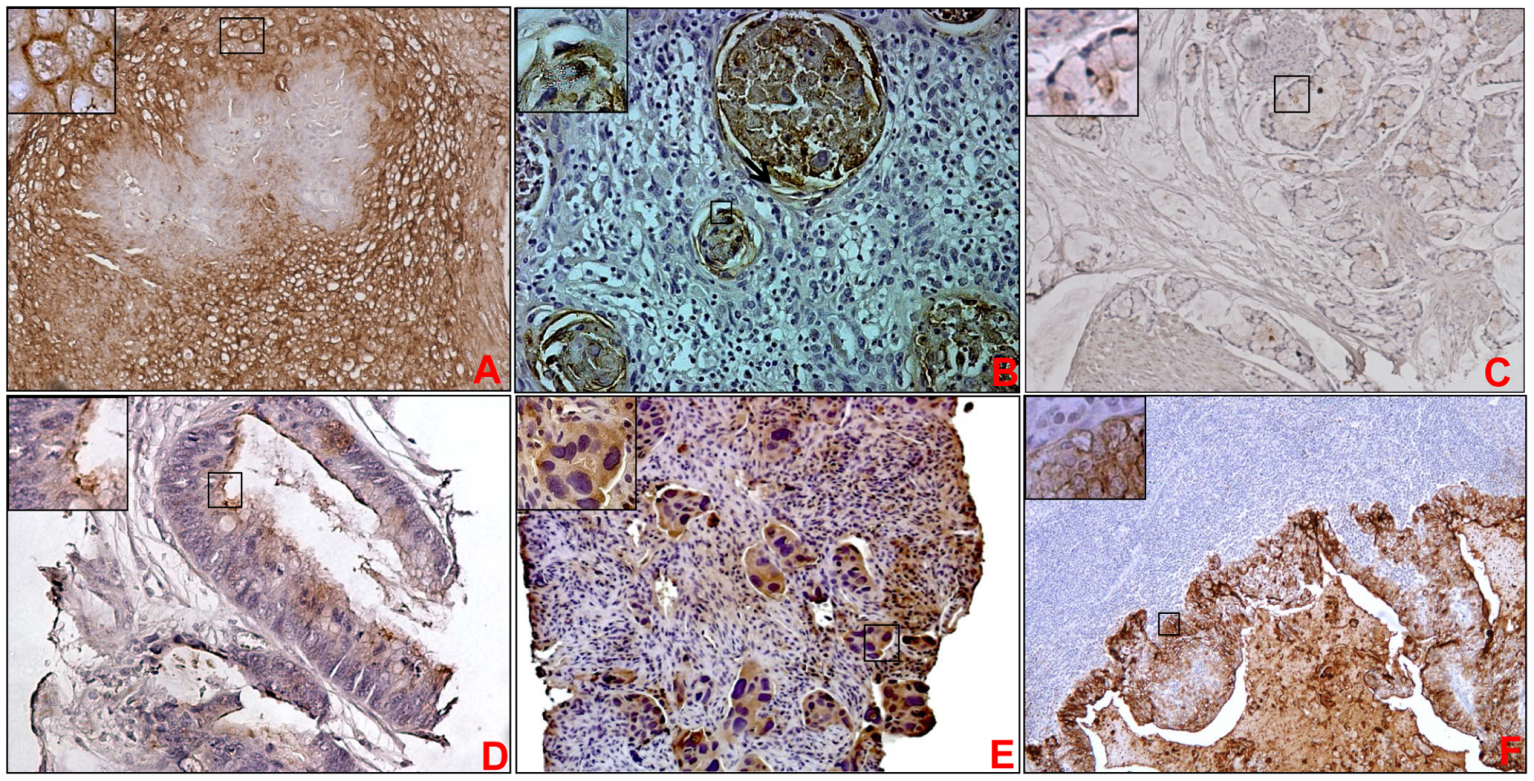
Expression of MUC1 in various bladder carcinoma phenotypes and metastases. Representative photomicrographs shown for MUC1 staining in squamous cell carcinoma (A), keratin pearls (B), adenocarcinoma (C) mucinous adenocarcinoma (D), metastasis to bone (E), metastatic urothelial carcinoma in lymph node (F). Strong membranous expression of MUC1 was observed squamous cell carcinoma (A) and also in keratin pearls within the squamous cell carcinoma (B). Both mucinous adenocarcinoma (C) and adenocarcinoma showed weak staining (D) for MUC1. Strong expression of MUC1 was observed in metastatic urothelial carcinoma in bone (E) and lymph node (F). High power view showed that MUC1 is localized to both cytoplasm and membrane in the metastases (inset of E&F).

MUC1 expression was observed in 66% (8/12) of metastases from a primary UC to various locations *i.e*. abdominal wall, back, bone, brain and lymph nodes (**Fig. 2E and F** **)**. In case of metastatic tissue sections (n = 28), absolute expression of MUC1 was observed in all metastatic cases with mean H-score of 1.91±0.15 and mean intensity of 2.72±0.45. In accordance with these results, no MUC1 expression was observed in normal lymph node area while metastatic cells within the lymph node have positive reactivity (**Fig. 2E** **)**. The observed MUC1 expression can be categorized into three different patterns luminal membrane staining (in the umbrella cell layer) only, luminal plus cytoplasmic staining (in intermediate and basal layers), or staining of only isolated cells or cell groups.

As seen in T**able 2**, significant difference in H-score of MUC1 staining was observed from grade 1 (0.51±0.1) to grade 2 tumors (1.62±0.155). Further H-score for MUC1 staining increased from grade 3 to grade 4 tumors.

**Table 2:** Variation of MUC1 expression with grades of urothelial carcinoma.

	Urothelial Carcinoma				
Grade	N	H-score		Intensity	
		Mean ±SE	Median	Mean ±SE	Median
**1**	34	0.51±0.1^a^	0.15	2.37±0.12 a	2.5
**2**	70	1.62±0.16 b	1.55	2.19±0.13	3.0
**3**	193	1.70±0.08	1.6	2.1±0.078	2
**4**	21	2.29±0.2^c^	3	2.8±0.08^c^	3

Standard error (SE);

a comparison Grade 1 vs. non-neoplastic urothelium (p≤0.005);

b p≤0.005 vs. Grade 1;

c p<0.05 vs. Grade 3.

#### MUC1 expression in Squamous Cell Carcinoma (SCC), adenocarcinoma and mucinous adenocarcinoma of the bladder

Moderate to intense staining was observed in SCC (N = 33, 65% of cases) with the mean H-score 2.33±0.16 (**Fig. 2A**), while others showed only mild positivity for MUC1 (N = 18 or 35%, mean H-score 0.66±0.11, mean intensity 1.3±0.11). Moderate staining for MUC1 was observed in keratin pearls which are distinctive feature of grade 1 squamous cell carcinoma (**Fig. 2B** **, depicted by arrow**). There was no significant difference in staining intensity between grade 2 (N = 6, mean H-score 2±0.3) and grade 3 SCC (N = 12, mean H-score 3.0±0.7). Of the 12 adenocarcinomas spots which were examined, 7 showed weak staining of MUC1 (**Fig. 2C**) while in others expression was completely absent. Majority of tissue spots from mucinous adenocarcinoma were mildly positive for MUC1 (mean H-score, 0.62±0.14) (**Fig. 2D**).

### MUC4 Expression Analyses during Malignant Neoplasm of Bladder

#### MUC4 expression in the non-neoplastic bladder urothelium

**Figure 3:**
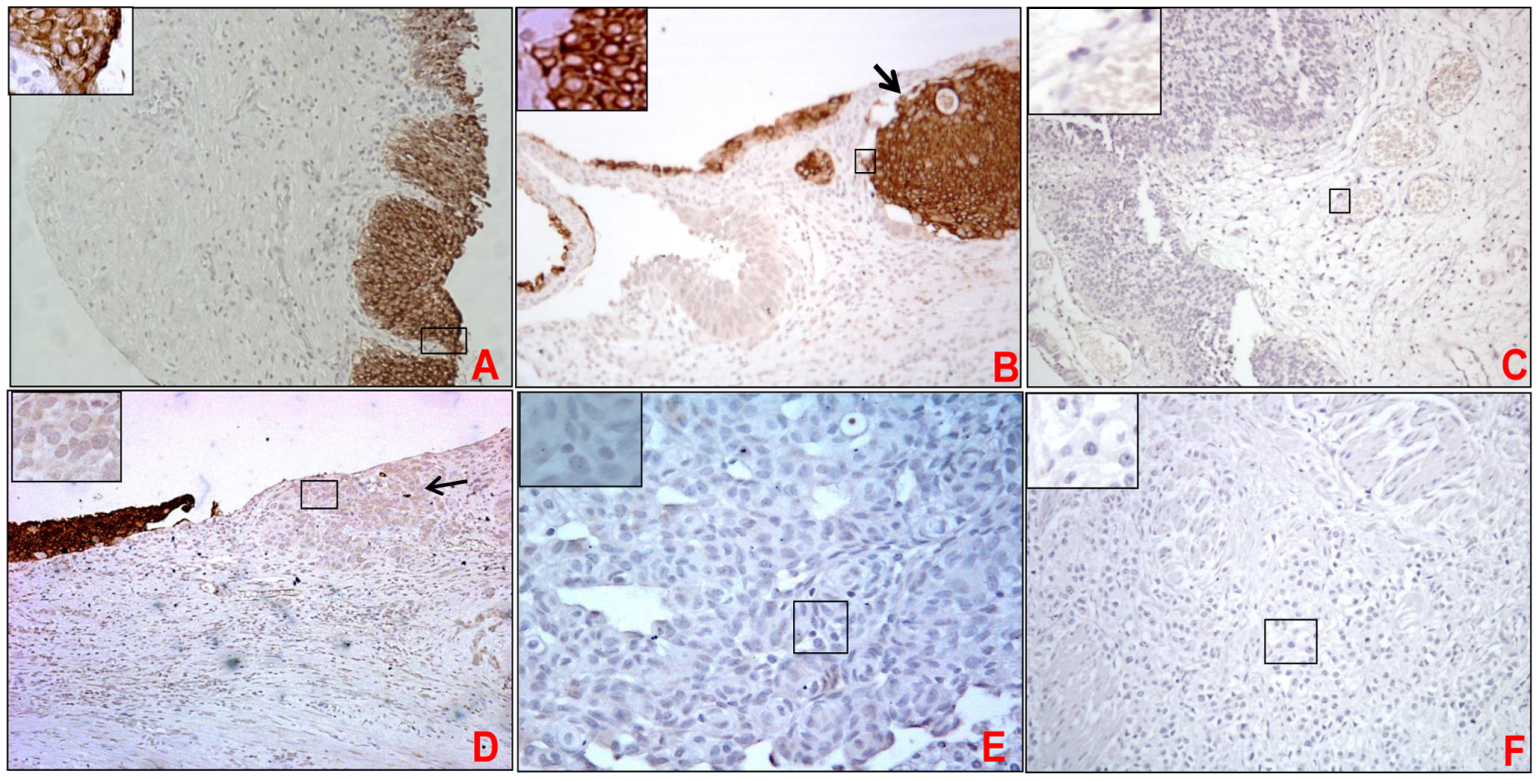
Expression of MUC4 in non-neoplastic bladder urothelium, papillary urothelial carcinoma, urothelial carcinoma in situ, invasive low and high grade urothelial carcinoma. Bladder tumor tissue sections and tissue microarray were probed with anti-MUC4 mAb (8G7) after non-specific blocking with horse serum. All sections were examined under microscope and the immunoreactivity was evaluated by reddish brown staining. Representative photomicrographs are shown for MUC4 stained normal non-neoplastic urothelium (A), von Brunn’s nests (B), non-invasive papillary urothelial carcinoma (C), urothelial carcinoma in situ (D), low (E) and high grade urothelial carcinoma (F). Strong positivity of MUC4 was observed in various layer bladder urothelium (A). Magnified image shows that distribution of MUC4 is both membranous and cytoplasmic. Cells in von Brunn’s nest cells showed strong membranous staining of MUC4 (B). Loss of MUC4 expression was observed during non-invasive papillary carcinoma (C), and carcinoma in situ (D). Both low and high grade urothelial carcinoma showed loss of MUC4 expression (E&F).

When analyzed in normal tissues (N = 14), high expression of MUC4 was observed in urothelium of the non-neoplastic bladder (**Fig. 3A**, mean intensity 2.54±0.21 and mean H-score 2.54±0.21) (**Table 3**). The expression of MUC4 (in the normal bladder urothelium) was observed both in the membrane and cytoplasm of epithelium cells with stronger expression on cell membranes than cytoplasm. However, in contrast to MUC1, the staining for MUC4 was uniform through all the layers of urothelium (**Fig. 3A**). Interestingly, a strong expression of MUC4 was observed even in invaginated aggregates of urothelial cells known as “von Brunn’s nests” (**Fig. 3B** **, depicted by arrow**).

**Table 3:** MUC4 expression in non-neoplastic urothelium and various malignant phenotypes of bladder.

Expression of MUC4 in Tissue Microarray (TMA)											
	Non-Neoplastic	Carcinoma in situ		Benign Hyperplasia		UC	Sarcoma		SCC		Adeno-carcinoma
**N**	14*	4		4		211	8		51		8
**Intensity score (Average ± SE)**	2.58±0.28	0		3		0.56±0.06	8		2.4±0.26^a^^,^ c		0.5±0.38^a^^,^ c
**Intensity (Median)**	3	0		3		0	0		2		0
**Intensity score (Range)**	0–3	–		–		0–3	0		0–3		0–3
**H-score (Mean ± SE)**	2.54±0.21	0		3		0.47±0.05^a^^,^ b	0		0.7±0.13 a ^,^ b		0.4±0.22
**H-score (Median)**	3	0		3		0	0		0.28		0
**H-score (Range)**	0–3	–		–		–	–		0–3		0–2
**Expression of MUC4 in Tissue Section**											
	**Benign Hyperplasia**		**Papillary carcinoma**		**Carcinoma in situ**			**UC**		**Metastatic UC**	
**N**	3		10		10			15		28	
**Intensity score (Mean ± SE)**	2.5±0.5^c^		0.8±0.41		0.3±0.1^d^			0.83±0.32		1.57±1.3	
**Intensity (Median)**	3		0		0			0		2	
**Intensity score (Range)**	0–3		0–3		0–3			0–3		0–3	
**H- score (Mean ± SE)**	0.96±0.63		0.31±0.39		0.12±0.06			0.4±0.18		0.73±0.92	
**H- score (Median)**	0.75		0		0			0		0.165	
**H- score (Range)**	0–3		0–3		0–2.4			0–2.4		0–3	

a comparison vs. non-neoplastic urothelium.

b p≤0.0001 vs. non-neoplastic urothelium.

c p≤0.005 vs. non-neoplastic urothelium.

d p<0.05 vs. non-neoplastic urothelium.

Urothelial carcinoma (UC); Squamous cell carcinoma (SCC); Standard error (SE).

*Non-neoplastic tissues combined from TMA and tissue sections.

#### MUC4 expression in non-invasive and invasive papillary urothelial carcinoma and urothelial Carcinoma In Situ (CIS)

Although limited cases (N = 6) of non-invasive papillary carcinoma were present in tissue spots, majority of them were completely negative (N = 4, intensity-score 0) while other have partial positivity (N = 2, mean intensity-score 2.5). Invasive papillary carcinoma (N = 2) showed complete loss of MUC4 expression (Intensity-score = 0). Further, complete loss of MUC4 expression was observed in both non-invasive (**Fig. 3C**) and invasive cases of papillary carcinoma (N = 10, mean intensity 0.8±0.41; mean H-score 0.31±0.39). MUC4 expression was significantly downregulated in CIS from TMA (N = 4, mean H-score 0, **table 3**) as well as in case of tissue sections (N = 10; mean intensity 0.3±0.1; p = 0.02 and mean H-score 0.12±0.06) compared to the non-neoplastic urothelium (**Fig. 3D**). Overall, loss of MUC4 expression was observed during invasive and non-invasive papillary carcinoma.

#### MUC4 expression in invasive urothelial carcinoma and metastasis

**Figure 4:**
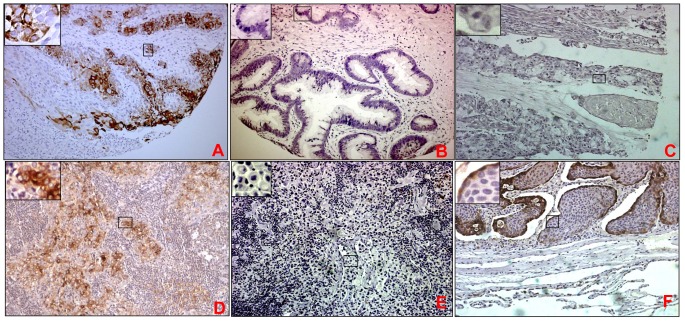
Expression of MUC4 in various bladder carcinoma phenotypes and metastases. Representative photomicrographs shown for MUC4 staining in squamous cell carcinoma (A), adenocarcinoma (B), mucinous adenocarcinoma (C) and metastatic urothelial carcinoma lymph node (D&E), and metastatic urothelial carcinoma lung (F). Squamous cell carcinoma cases showed moderate expression of MUC4 while MUC4 was found to be absent in adenocarcinoma. Focal to moderate expression of MUC4 was observed in metastatic urothelial carcinoma to the various organs. High power view showed that staining pattern is both membranous and cytoplasmic during metastasis.

In majority of the cases, focal expression of MUC4 was observed in urothelial carcinoma (N = 122 or 58%, mean H-score, mean intensity 0) whereas other cases showed mild positivity (N = 89 or 42% cases, mean H-score 0.68±0.14, mean intensity 0.91±0.19). Overall, the loss of MUC4 expression was observed in both low and high grade invasive urothelial carcinoma (**Fig. 3E and 3F**). MUC4 expression was significantly down-regulated in carcinoma cells compared to the non-neoplastic urothelium (mean H-score in the non-neoplastic bladder urothelium and UC being 2.54±0.21 [p<0.0001], and 0.47±0.05 respectively) (data from TMA). Interestingly, in comparison to urothelial carcinoma that lacks the expression of MUC4, higher percentage of metastatic cases were found to be positive for MUC4. Among all (N = 28) metastatic cases, 57% cases (N = 16) demonstrated weak to mild MUC4 staining (mean H-score 1.27±0.27) (**Fig. 4D–F**) while none to focal expression was observed in 43% of cases (N = 12; mean intensity = 0 and mean H-score 0.025±0.02). Both cytoplasmic and membrane staining of MUC4 was observed in various metastatic cases (**4D–F**).

As seen in **Table 4**, no significant difference in H-score of MUC4 staining was observed from grade 2 (0.66±0.17) to grade 4 tumors (0.58**±**0.19).

**Table 4:** Variation of MUC4 expression with grades of Urothelial Carcinoma.

	Urothelial Carcinoma				
Grade	N	H- score		Mean Intensity(±SE)	
		Mean (±SE)	Median	Mean (±SE)	Median
**2**	33	0.66±0.17	0.5	0.68±0.18	0.5
**3**	118	0.54±0.07	0.25	0.68±0.18	0.5
**4**	19	0.58**±**0.19	0.58	0.61±0.21	0.6

#### MUC4 expression in Squamous Cell Carcinoma (SCC) and adenocarcinoma of the bladder

Of the 51 SCC spots which were examined (in the TMA), focal positivity was observed in 38% cases (N = 19, mean H-score 0.061±0.009), mild positivity in 42% cases (N = 21, mean H-score 0.59±0.09) and intense reactivity in 20% cases (N = 10, mean H-score 2.24±0.13) (**Fig. 4A**). Strong membranous staining was observed in majority of cells with few cells showing cytoplasmic staining. In MUC4 positive cases, all invasive foci of SCC within the lamina propria showed uniform staining (**Fig. 4A**). No significant co-relation could be assessed between MUC4 expression and grade of squamous cell carcinoma due to limited information on tumor grade of cases. Although the number of tissue sections were limited for adenocarcinoma (N = 8), no or focal MUC4 reactivity was observed (mean H-score 0.4±0.22) **(** **Fig. 4B**). Similarly, mucinous adenocarcinomas were also found to negative for MUC4 expression (**Fig. 4C**).

### Expression of MUC1 and MUC4 in Bladder Carcinoma Cell Lines

**Figure 5:**
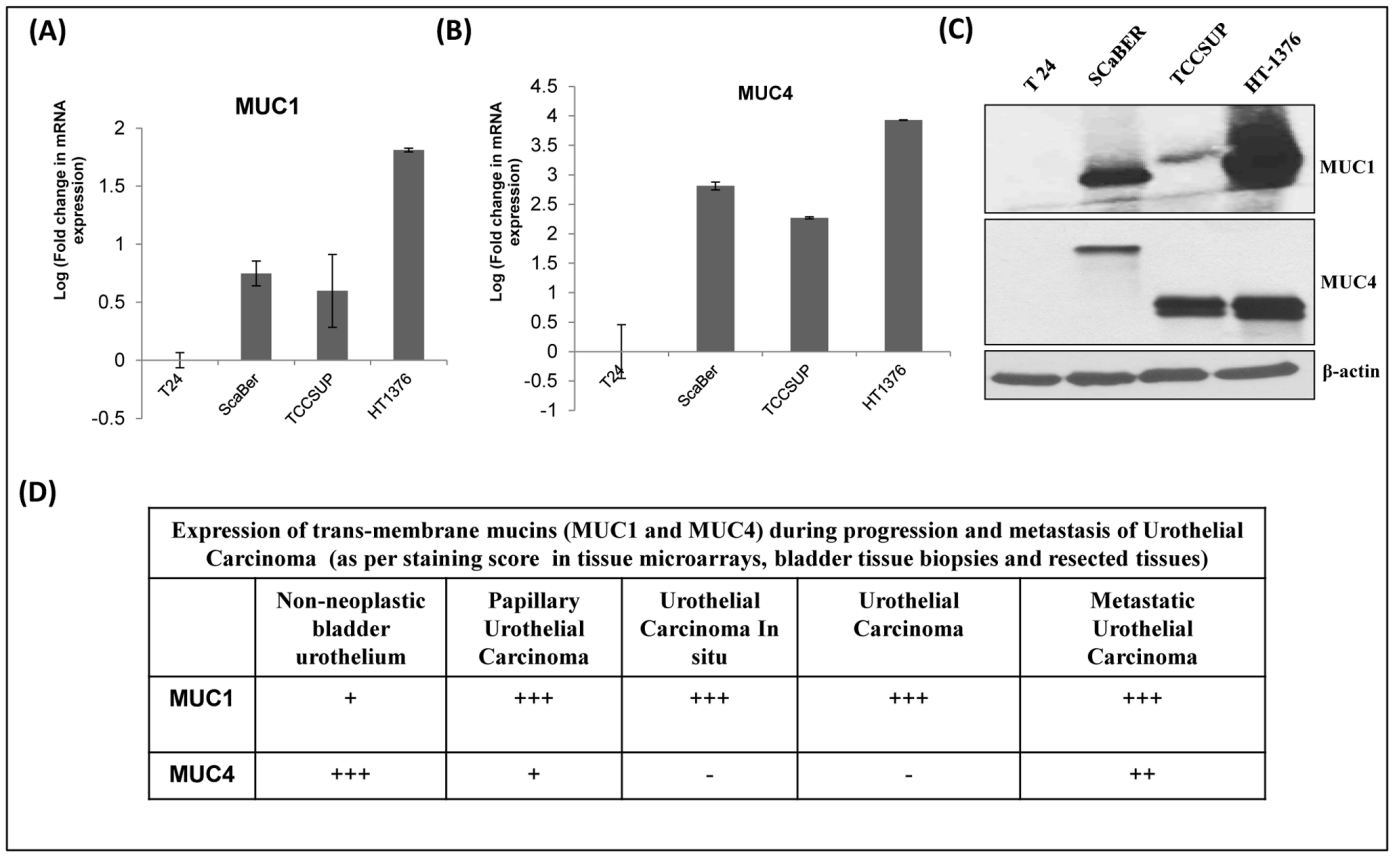
Expression analysis of transmembrane mucins MUC1 and MUC4 at RNA and protein level in various bladder carcinoma cell lines and tabular representation of mucin expression as observed during tissue staining in the urothelial carcinoma progression. Quantitative RT-PCR analyses of MUC1 (A) and MUC4 (B) showed that all bladder carcinoma cell line except T24 expresses MUC1 and MUC4. Highest expression of both MUC1 and MUC4 was observed in tumor cell line HT1376. For expression analyses of mucin MUC1 and MUC4 at protein level, the protein lysates from all the bladder carcinoma cell lines were resolved on 2% agarose gel and probed with anti-MUC1 (HMFG2) and anti-MUC4 (8G7) antibodies (Panel C). β-actin resolved on 10% SDS-PAGE was used as a loading control (Panel C). No expression of any mucin MUC1 and MUC4 was observed in T24 while other cell lines expressed both the mucins. (D) Tabular representations of MUC1 and MUC4 expression during bladder carcinoma progression. Significant changes in expression of both MUC1 and MUC4 were observed during progression of urothelial carcinoma. Progressive increase in MUC1 expression was observed through various stages and grades of bladder cancer whereas the expression of MUC4 declined with disease progression. In a subset of metastatic cases of urothelial carcinoma, the expression of MUC4 was detected.

After analyzing the expression of mucins in various bladder pathologies by immunohistochemistry, the expression of MUC1 and MUC4 was analyzed at mRNA and protein levels in different bladder carcinoma cell lines (**Fig. 5** **, panel A–C**). The panel included four bladder carcinoma cell lines-T24, TCCSUP, HT1376 and Scaber. Quantitative RT-PCR for MUC1 and MUC4 revealed that all the cell lines except T24 expressed MUC1 and MUC4 (**Fig. 5A&B** **)**. Immunoblotting studies using anti-MUC1 antibody (HMFG2) and anti-MUC4 antibody (8G7) indicated differential expression of MUC1 and MUC4 in TCCSUP, HT1376 and Scaber cell lines (**Fig. 5C**). No expression of MUC1 and MUC4 was observed in T24 bladder carcinoma cell line. Further, different glycoforms of MUC4 were observed in squamous cell carcinoma cell line in comparison to TCC cell lines.

Overall, mucin MUC1 and MUC4 analyses in bladder cancer TMA and tissue sections indicated that the expression of MUC1 is increased while that of MUC4 decreased in UC compared to the normal non-neoplastic urothelium. Expression of both MUC1 and MUC4, however, are significantly higher in urothelial carcinoma metastatic cases compared to localized UC (**Fig. 5D**).

## Discussion

Aberrant expression, localization and glycosylation of mucins are characteristic events of multiple malignancies (pancreatic, ovarian, prostate and gastric, lung, and breast). Among various family members, MUC1 and MUC4 are implicated in tumor growth, intracellular and extracellular signaling, tumor–stromal interactions, metastasis, and resistance to chemotherapeutic agents and in immunity [10], [12]. Comprehensive information regarding expression of these mucins is missing in urothelial carcinoma. The present study examined the expression of TM mucins MUC1 and MUC4 in bladder tissue microarray, tissue sections and bladder carcinoma cell lines. While aberrant changes were observed in expression and localization pattern of MUC1 between benign and malignant cases, significant downregulation of MUC4 was observed during urothelial carcinoma compared to normal and/or benign bladder tissues.

MUC1 is a membrane-bound *O*-glycoprotein that is expressed at the basal level in most epithelial cells [21]. Deregulated expression of MUC1 is a prominent characteristic of various types of cancers and inflammatory diseases. Overexpression and knockdown studies of MUC1, revealed that it leads to increased tumorigenicity and anti-adhesive properties in number of systems including cancer cell lines of pancreas, breast, and myeloma, as well as in MUC1 transgenic model of human breast cancer and MUC1-transfected 3Y1 rat fibroblasts [12], [22]–[25]. In the present study, immunohistochemical analyses of MUC1 by HMFG2 antibody revealed that in benign bladder urothelium, MUC1 expression was either restricted to umbrella cells which is observed in majority of the cases, or it forms a sheath over transitional epithelium as seen in rare cases. Further, the superficial layer of bladder urothelium also shows a strong positivity for MUC1 expression in certain cases. Our observation was in accordance with Simms et al study where presence of MUC1 was observed in apical layer of transitional epithelium using anti-MUC1 C595 antibody [17]. Further, Patriarca et al also observed the expression of MUC1 in apical pole of umbrella cells of normal urothelium [16].

In case of papillary carcinoma, we observed moderate to strong staining of MUC1 in the apical layer in majority of the tissue sections. Further, the expression of MUC1 was observed in luminal as well as in intermediate and basal layers of uroepithelium. Interestingly, some of the epithelial cells showed much higher staining for MUC1 in comparison to cells in the vicinity in some cases of papillary carcinoma. It might account for the speculation that aberrant changes in MUC1 expression pattern are due to loss of polarization. In earlier studies, it is suggested that MUC1 plays a significant role in lumen formation, and has an inhibitory role in the cell to stromal interaction. From these studies, it is conceivable that it is a key factor in the detachment of cells from stroma, allowing for the dissection of the connective tissue and easing the spread of cell. Elazeez et al observed that 74% of cases of papillary transitional cell carcinoma were positive for MUC1. Also, the same study demonstrated an increased expression of MUC1 with increasing grade of papillary carcinoma *i.e.* 37.5% of grade 1 cases, 75% of grade 2 cases and 88.9% of grade 3 cases and this difference in expression was statistically significant (P<0.01) [15].

In case of urothelial carcinoma, expression of MUC1 showed varied trend, starting from lack of expression (N = 38 or 12%), focal reactivity (N = 12 or 4%, mean H-score 0.057±0.01) to moderate (N = 147 or 46%, mean H-score 1.02±0.05) and intense immunoreactivity (N = 117 or 37%, mean H-score of 2.86±0.02). The H-score for MUC1 staining increased from grade 1 to grade 2 tumor (**Table 3**). Further, MUC1 expression was quite high in 66% (8/12) of metastases cases of urothelial carcinoma to various locations *i.e.* abdominal wall, back and left frontal lobe of brain while all the metastases cases from biopsies and resected tissues (N = 28) were strongly positive for MUC1 expression (mean intensity 2.72±0.45). MUC1 is thought to provide anti-adhesive properties by counteracting the interactions between adhesion molecules such as integrin and E-cadherin [26]. Interestingly, MUC1 overexpression under *in vitro* conditions has been shown to reduces adhesion between adjacent cells and between cells and extracellular matrix (ECM) by mediating binding to some molecular ligands and blocking binding with other ligands [27]. Similar to these observations, we observed strong expression of MUC1 towards luminal region of invasive carcinoma cases.

MUC4 is another heavily glycosylated high molecular weight transmembrane glycoprotein. Despite several studies on the oncogenic potential of MUC4 in various cancers, there is no detailed study on the expression of MUC4 in bladder pathology. In the present study, we examined the expression pattern of MUC4 in non-neoplastic bladder urothelium and various phenotypes of malignant bladder. In the non-neoplastic bladder urothelium, intense staining of MUC4 was observed in all the layers of urothelium. In contrast to MUC1, staining of MUC4 was uniform throughout all the layers of urothelium. Interestingly, intense expression of MUC4 was observed in invaginated aggregates of urothelial cells known as von Brunn’s nest. In case of papillary urothelial carcinoma, loss of MUC4 expression was observed. Analyses of MUC4 in CIS cases revealed the loss of MUC4 mucin during tumorprogression. Further MUC4 expression was either absent or present focally in low grade and high grade invasive carcinoma cases from tissues. Additionally during the study, low expression of MUC4 in comparison to non-neoplastic bladder urothelium was observed in SCC and adenocarcinoma. Since the loss of MUC4 expression was observed in non-invasive papillary UC and urothelial CIS cases, the present study suggests that loss of MUC4 might be one of the early events during development of urothelial carcinoma. Similar to our study, Singh et al observed the loss of MUC4 during development of prostate cancer [7]. During the study, they observed that the expression level of MUC4 was much lower in prostatic adenocarcinoma tissue (CaP) compared to adjacent normal/benign prostate tissue (N/BPH). Treatment of prostate cancer cell lines with inhibitor of histone deacetylases and DNA methyl transferases lead to increased expression of MUC4. Singh et al, suggested the epigenetic mechanism might be regulating the MUC4 expression during pathogenesis of prostate cancer. Further studies need to be carried out in urothelial carcinoma to decipher the mechanism of MUC4 downregulation. In summary, intense expression of MUC4 was observed in normal epithelium and its progressive loss in carcinoma in situ/papillary carcinoma stage which was followed by complete loss during high and low grade invasive carcinoma (**Fig. 5D**). In our study, in limited cases, we observed that expression of MUC4 comes back during metastasis, however further studies are warranted to confirm this observation.

### Conclusions

In conclusion, our findings suggest that both the pattern and level of mucin expression is significantly associated with the type of urothelial carcinoma. With regard to MUC1, aberrant changes were observed both in expression and localization pattern while significant loss of MUC4 expression was observed with progression of UC. Since the loss of MUC4 expression is observed at very early stages, our study indicates that MUC4 loss might be of diagnostic relevance for early detection of urothelial carcinoma. Further in metastatic cases, we observed revival of MUC4 expression. Immunohistochemistry for MUC4 may be a useful adjunct to morphological assessment of difficult cases in distinguishing urothelial CIS/carcinoma from its benign mimics. Further studies are warranted to decipher the molecular mechanism and effect of MUC4 loss on development of bladder carcinoma.
